# A Social Network Analysis of Tweets Related to Mandatory COVID-19 Vaccination in Poland

**DOI:** 10.3390/vaccines10050750

**Published:** 2022-05-10

**Authors:** Rafał Olszowski, Michał Zabdyr-Jamróz, Sebastian Baran, Piotr Pięta, Wasim Ahmed

**Affiliations:** 1 Faculty of Humanities, AGH University of Science and Technology, Gramatyka 8a, 30-071 Kraków, Poland; pipieta@agh.edu.pl; 2 Center for Collective Intelligence, Massachusetts Institute of Technology, 245 First Street, E94, Cambridge, MA 02142, USA; 3 Faculty of Health Sciences, Institute of Public Health, Jagiellonian University Medical College, Skawinska 8 Street, 31-066 Kraków, Poland; michal.zabdyr-jamroz@uj.edu.pl; 4 Department of Mathematics, Cracow University of Economics, Rakowicka 27, 31-510 Kraków, Poland; sebastian.baran@uek.krakow.pl; 5 Stirling University Management School, University of Stirling, Stirling FK9 4LA, UK; wasim.ahmed@stir.ac.uk

**Keywords:** COVID-19, mandatory vaccination, vaccination hesitancy, social network analysis, social media, Twitter debate, Poland

## Abstract

Poland’s efforts to combat COVID-19 were hindered by endemic vaccination hesitancy and the prevalence of opponents to pandemic restrictions. In this environment, the policy of a COVID-19 vaccination mandate faces strong resistance in the public debate. Exploring the discourse around this resistance could help uncover the motives and develop an understanding of vaccination hesitancy in Poland. This paper aims to conduct a social network analysis and content analysis of Twitter discussions around the intention of the Polish Ministry of Health to introduce mandatory vaccinations for COVID-19. Twitter was chosen as a platform to study because of the critical role it played during the global health crisis. Twitter data were retrieved from 26 July to 9 December 2021 through the API v2 for Academic Research, and analysed using NodeXL and Gephi. When conducting social network analysis, nodes were ranked by their betweenness centrality. Clustering analysis with the Clauset–Newman–Moore algorithm revealed two important groups of users: advocates and opponents of mandatory vaccination. The temporal trends of tweets, the most used hashtags, the sentiment expressed in the most popular tweets, and correlations with epidemiological data were also studied. The results reveal a substantial degree of polarisation, a high intensity of the discussion, and a high degree of involvement of Twitter users. Vaccination mandate advocates were consistently more numerous, but less engaged and less mobilised to “preach” their own stances. Vaccination mandate opponents were vocal and more mobilised to participate: either as original authors or as information diffusers. Our research leads to the conclusion that systematic monitoring of the public debate on vaccines is essential not only in counteracting misinformation, but also in crafting evidence-based as well as emotionally motivating narratives.

## 1. Introduction

In late 2019, in the Chinese city of Wuhan, a new disease was reported and identified as COVID-19 (coronavirus disease 2019) with its pathogen SARS-CoV-2 [1]. In 2020, it became an international public health emergency, and in March 2020, the World Health Organization (WHO) declared the COVID-19 outbreak as a pandemic [2]. As of 13 December 2020, WHO reported over 269 million confirmed cases of COVID-19, with over 5.3 million deaths globally [3].

Initially, the only response measures available on a population scale for combating the pandemic (aside from life-saving medical interventions and the usual epidemic procedures: isolations, quarantines, testing, contact-tracing) were non-pharmaceutical interventions (NPIs): lockdowns, restrictions of movement and social gatherings, face masking, physical distancing [4]. However, NPIs—despite their significant merits—are not without their adverse direct and indirect side effects [5]. This includes limited access to healthcare services, especially for low-income people, and aggravating other health problems [6]. Lockdowns and other restrictions strongly increased stress and socio-economic anxieties, and generally resulted in a net loss in happiness [7]. The result was a heated public and scholarly debate about the validity of various pandemic strategies [8]. Thus, the greatest hope for combating the pandemic—and avoiding controversies about lockdowns—was the development and widespread use of COVID-19 vaccines—a tool that was intended to play a crucial role in the prevention and eradication of multiple dangerous infectious diseases [9].

The looming threat to the COVID-19 vaccination programme was the prevalence—preceding the pandemic itself—of anti-vaccination movements and vaccination hesitancy [10,11,12]. Vaccination hesitancy is defined by WHO as “delay in acceptance or refusal of safe vaccines despite availability of vaccine services” [13], and has a variety of determining factors, including the context and communication—especially lack of trust in political and scientific institutions, and biased information sources [14].

Despite the relatively swift development of multiple COVID-19 vaccines, together with the accumulation of evidence of their effectiveness [15] and safety [16], further controversies started arising—most notably about the access to vaccines in lower-income countries (e.g., debate over vaccines patents) [17,18]. The public discussion on COVID-19 vaccines was added to the lockdown controversy, and fuelled vaccination hesitancy. During the pandemic, a “new front” for medical and public health professionals emerged: the COVID-19 “infodemic”. An infodemic is broadly defined as “an excessive amount of information concerning a problem such that the solution is made more difficult. The end result is that an anxious public finds it difficult to distinguish between evidence-based information and a broad range of unreliable misinformation” [19].

The COVID-19 infodemic occurred in the “post-truth world”—in an environment particularly challenging for science communicators [20,21], with the proliferation of conspiracy theories and overflowing of “fake news” [22]—misinformation, disinformation, and malinformation [23]. Pre-pandemic studies in the US showed that the popularity of medical conspiracy theories (e.g., regarding cancer cures, vaccines, and cell phones) correlated with problematic health behaviours, such as greater use of alternative medicine and the avoidance of vaccination [24]. These conclusions are corroborated by the results of studies on the anti-vaccine movement and COVID-19 negationism, as well as public health campaigns in Spanish social media discourse [25,26].

Poland was also affected by the pandemic, with 2357 COVID-19 deaths per 1 million [27], a cumulative number of over 3.8 million cases, and over 88 thousand deaths [28]. In 2020 alone, that meant 67 thousand excess deaths [29]. Poland implemented anti-COVID-19 basic public health measures relatively quickly during the first wave of the pandemic—introducing lockdowns, mask mandates, remote work and schooling, etc. [30]. However, later on, the country faced growing difficulties in pandemic emergency management, among others, due to endemic problems in the Polish healthcare system and severe medical personnel deficits [31]. Despite these difficulties, the initial rollout and uptake of COVID-19 vaccines was promising. Later, however, vaccination reached its peak—limited by hesitancy among the general populace. As of 5 December 2021, 39.2 million vaccine doses have been administered [28], and approximately 53% of the population received two doses of the vaccine [32]. Between January and April 2021, almost one-third of adult inhabitants of Poland declared an unwillingness to vaccinate against COVID-19. Two-thirds of those unwilling stated that they are concerned about the side effects of the vaccine [33].

Studies on COVID-19 vaccination hesitancy indicate a variety of notable factors, but confirm that the primary rationale given for vaccination hesitancy is “fear of the side effects of the COVID-19 vaccine” (allergic reactions or other adverse events) [33,34]. This research also shows that changes in the attitudes of inhabitants of Poland might occur alongside certain discursive events, i.e., events, statements, or decisions that invoke notable activity in the public discourse [33]. This might be particularly relevant when it comes to periods of rising levels of public debate over different forms of COVID-19 vaccination mandates. Studies show that mandatory vaccination paired with notable financial sanctions were associated with higher vaccination coverage and reduced incidence of dangerous infectious diseases [35]. Multiple ethical justifications have been delivered to support vaccination mandates [36]. However, public and political debate over the legitimacy of this policy decision is far from being settled. Thus, it is relevant to understand what type of discourse emerges concerning the issue of vaccines in the context of proposals for making them obligatory.

This paper aims to conduct a social network analysis and content analysis of Twitter discussions around the intention of the Polish Ministry of Health to introduce mandatory vaccinations for COVID-19. Twitter, as an environment for researching the information flow, was selected because of its critical role in cascading medical information, and misinformation, during the COVID-19 pandemic [37].

The study set out to address the following research questions:

RQ 1: What was the response on Twitter around the announcement of introducing mandatory vaccinations for COVID-19 in Poland, and what network shape emerged? What features characterised the key groups of advocates and opponents of mandatory vaccinations, and how was their activity correlated with official epidemiological data concerning the COVID-19 pandemic?

RQ2: What was the general sentiment concerning mandatory vaccinations for COVID-19 expressed in the most popular tweets? How is this sentiment correlated with official epidemiological data concerning the COVID-19 pandemic?

The following section discusses the materials and methods used during the research. In Section three, the social network analysis, the review of events associated with Twitter users’ activity, and measures of correlation of Twitter users’ activity and epidemiological data, are evaluated, followed by a review of the influential users, and an analysis of the most popular tweets. Correlations between sentiment in the top-10 tweets and the epidemiological data, as well as correlations between the overall network metrics and the epidemiological data, are also presented. Conclusions and recommendations for future research are provided at the end of the paper.

## 2. Materials and Methods

### 2.1. Data Collection

Extraction of unstructured data from Twitter has been performed using R scripts through the Application Programming Interface (API) v2 for Academic Research, which allows researchers the ability to retrieve tweets from the entire Twitter archive. The tweet selection criteria were (i) tweets published in the Polish language, (ii) tweets containing the keywords “obowiązkowe szczepienia” (“mandatory vaccination”), “przymusowe szczepienia” (“compulsory vaccination”), “obowiązek szczepień” (“obligation to vaccinate”), “przymus szczepień” (“compulsion to vaccinate”), and (iii) tweets that were published between 26 July 2021 (12:00 a.m. CET) and 11 December 2021 (11:59 p.m. CET). The time frame selected for this study is related to the date that the Medical Council of the Polish Prime Minister officially recommended the introduction of mandatory vaccinations (2 July 2021) [38] and the date that the Minister of Health of the Republic of Poland announced the introduction of vaccination obligations for selected professional groups from 1 March 2022 (7 December 2021) [39]. In the case of the end date of data collection, we took into account an additional two days after 7 December to collect opinions regarding the above-mentioned announcement. The Twitter users included in the data analysis were those who sent tweets with the above-mentioned characteristics during the predefined period. Unverified users were also included, as one of the objectives of the study was to analyse message dissemination.

A total number of 71,908 tweets, retweets, and replies were collected. These were then extracted, imported into the NodeXL software (Social Media Research Foundation, Redwood City, CA, USA), and analysed using social network analysis, as described in the next section. This allowed a network graph to be created. The dataset contained 21,779 Twitter users (including users who were replied to or mentioned in tweets).

In addition, we collected official statistics from the Ministry of Health of the Republic of Poland on the number of vaccinations carried out for each day covered by our analysis [40]. The second dataset, obtained from the same source, consists of various epidemiological data related to the SARS-CoV-2 pandemic: new cases of infection, new cases per 10,000 residents of the country, deaths caused by COVID-19 only, deaths caused by COVID-19 and comorbidities, number of people in quarantine, number of positive tests performed, number of negative tests performed, number of convalescents [41].

### 2.2. Social Network Analysis

A number of techniques were used to analyse the data. The software NodeXL was used to generate network metrics, such as betweenness centrality and network clusters, with a validated methodology used in previous research [42,43,44,45]. This analysis was then imported into the Gephi software in order to produce a visualisation of the NodeXL metrics, using the Force Atlas 2 layout algorithm [46]. The size of the nodes in the graph were ranked by their betweenness centrality score (BCS) [47]. The BCS measures the influence of a vertex over the flow of information between all other vertices (under the assumption that information flows over the shortest paths among them). The adopted method was based on drawing edges between each tweet and each connected “retweet” or “replies-to” relationship in a tweet, as well as each “mentions” relationship. A self-loop edge was drawn for each tweet that was not connected in any way. The graph’s vertices were grouped by cluster using the Clauset–Newman–Moore algorithm [48]. We assumed that the minimum size of the group that allows for the analysis was 10 users. In terms of understanding the network graph, the results of this study were derived using the methodology developed in previous research [45]; this revealed that Twitter topics might follow six network shapes and structures: polarised crowds, community clusters, broadcast networks, tight crowds, brand clusters, and support networks [44]. We then examined the time series of users’ activities, the most popular hashtags, the most influential users, and the top mentioned users.

Sentiment analysis, also known as opinion mining, was used to help us understand how people expressed their opinions, attitudes, and emotions toward the topic of mandatory vaccination [49]. For this study, sentiment analysis was deemed most appropriate to provide further insight into previous academic research, with a similar methodological approach to the use of social media during the pandemic [50]. Due to the fact that none of the methods of automatic content analysis based on natural language processing produced satisfactory results, we decided to analyse the tweets manually [51]. In addition to the aggregate analysis for all tweets in our database, we divided the entire studied sample into the one-week periods in order to capture opinion changes over time. Then, for each of these periods, we prepared a ranking of the top-10 most popular tweets in terms of the number of retweets made. A semantic analysis was performed for all these tweets. Coding categories were created by exploring the data, and the extracted sample was analysed and coded. The coding was confirmed by two authors, and any disagreements were discussed and resolved, which led to a 100% agreement. In addition, for each of the periods, we examined changes in social network properties, such as maximum and average geodesic distance, graph density, and modularity.

We also studied how the most important metrics used for overall social network analysis changed in the period we were interested in. We mainly examined the metrics related to collective intelligence emerging in the debate [52]: graph density, i.e., the number of possible or potential edges in the graph over the number of actual connections [53]; modularity, i.e., measure of the fitness of the groups that were created in a clustered network; maximum and average geodesic distance, i.e., the measurement of the distance between the vertices in a graph [54]; reciprocated vertex pair ratio, i.e., the percentage of vertices that have a reciprocal relationship; and reciprocated edge ratio, i.e., the percentage of edges that have a reciprocal relationship [43]. The NodeXL software was used to calculate the above-mentioned metrics.

Finally, we conducted a statistical analysis revealing the dependencies between the obtained data and the official epidemiological data provided by the Ministry of Health of the Republic of Poland. This made it possible to analyse the relationship between opinions expressed in social media and pandemic-related real-world phenomena.

## 3. Results

### 3.1. Social Network Analysis

**Figure 1:**
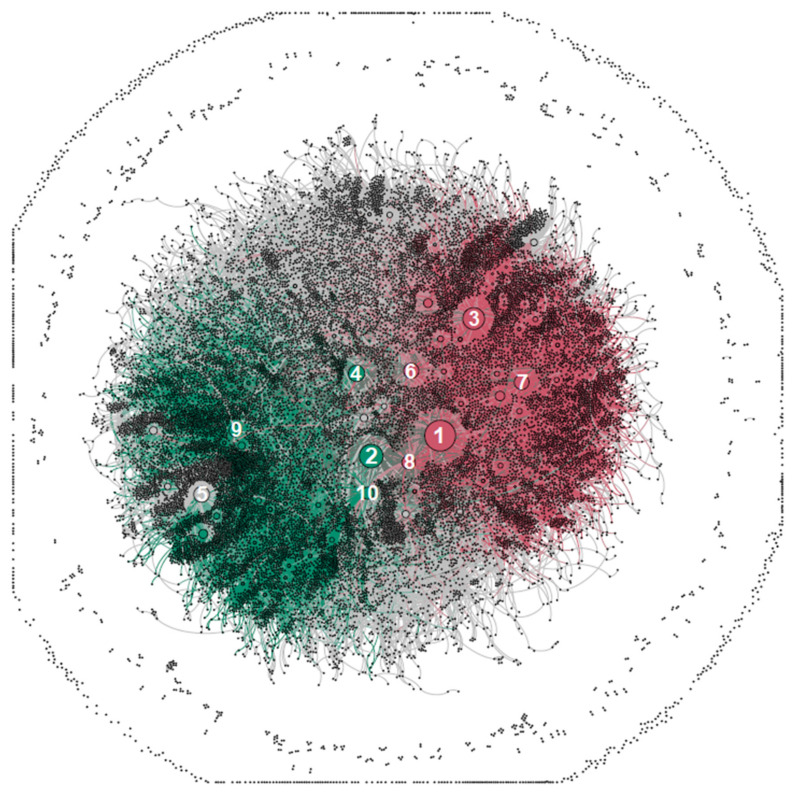
Social network graph of 21,779 Twitter users debating mandatory COVID-19 vaccination in Poland between 26 July 2021 and 9 December 2021. The two largest groups of users distinguished by the clustering algorithm are G1, marked with dark green (vaccination mandate advocates) and G2, marked with dark pink (vaccination mandate opponents). The users that did not belong to either of these two groups are visualised in grey. The 10 most influential users according to the betweenness centrality (BC) score are numbered from 1 to 10. The larger the nodes, the greater the BC score, and thus the more critical its position on the graph. The nodes on the outskirts of the graph, not connected with any other, belong to the isolates group, i.e., the users who sent tweets that did not contain mentions.

Figure 1 presents the Twitter users in social network graph clusters. Each node represents a user, and each line between them represents an edge. The sizes of the nodes are ranked by their betweenness centrality score (BCS) [47], which measures the influence of a vertex over the flow of information between all other vertices, under the assumption that information flows over the shortest paths among them. The clustering algorithm allowed for the identification of 27 groups meeting the assumed criteria. The graph, in particular, highlights the two most important clusters. The group visualised in dark green (G1) is the largest cluster of the network, consisting of 6520 users, which is 29.94% of the entire sample. The group visualised in dark pink (G2) is the second-largest group, consisting of 5930 users, which is 27.23% of the network.

Moreover, we note that the group G5 containing 777 nodes consists of isolates, i.e., the users who sent tweets that did not include mentions. This group is visible in the graph as a ring of unconnected nodes on the outskirts of the visualisation. Compared to the other social network debates that were the subject of similar studies, the relatively low position of the isolates group is noticeable. In many studies [42,45], isolates are the most numerous or second-most numerous group. None of the remaining 25 groups were greater than 14% of the sample, so they are collectively visualised in grey; in the first stage of our analysis, we focused on G1 and G2 as the most important clusters.

The first analysis we conducted was to answer the question of how many users had a profile related to a medical or scientific background. It should be emphasised that the vast majority of Twitter users do not provide information about their profession in the description of their accounts. However, we searched our user database for several variants of the following keywords: “physician”, “medical”, “doctor”, “professor”, “researcher”, “scientist”, etc. In our entire sample, we were able to identify only 271 users related to a medical or scientific background, which constituted 1.2% of all users. Moreover, in the G1 cluster, users of this type constituted 1.7% of the group (109 persons), and in the G2 cluster, they constituted only 0.7% of the group (45 persons).

The NodeXL software allowed us to cluster users into several groups based on the relationships between them; in particular, mentions, retweets, and replies [55]. The two most important user groups, i.e., G1 and G2, were of similar size and, as we prove below, presented highly polarised opinions. In general, the G1 group consisted mainly of vaccination mandate supporters, while the G2 group was comprised mainly of its opponents.

The nature of these groups becomes apparent when we compare their most popular hashtags. Table 1 below presents the most frequently used hashtags in group G1. As we can see, “covid19” hashtag (*n* = 1039), as well as “szczepimysie” (Eng. “we vaccinate”, *n* = 520) were the most popular hashtags. The other popular slogans in this group were the general references to the COVID-19 pandemic: “koronawirus”, “corona”, and “covid_19”. The hashtag “dworczyk” is related to Michał Dworczyk, the government spokesman responsible, inter alia, for communicating pandemic policies. “Pis” states for the political party Prawo i Sprawiedliwość (Eng. Law and Justice), which forms the government in Poland and is responsible for pandemic restrictions. The hashtag translated as “we vaccinate” appears twice because of the different forms of spelling of this slogan in Polish. “We vaccinate” is the slogan for COVID-19 vaccination proponents. An interesting fact is the appearance of the German term “impfpflicht”, also meaning the mandatory vaccination. The popularity of this hashtag is related to the frequent citation of tweets calling for the introduction of compulsory vaccinations in Germany and Austria. The conducted hashtag review and tweet content review (described in Section 3.5) allowed us to define the nature of the G1 group as supporters of the introduction of the vaccination mandate.

As we can see in Table 2, in the group G2, the most popular hashtag is “stopsegregacjisanitarnej” (Eng. “stop sanitary segregation”, *n* = 2090), which was a slogan used to protest COVID-19 restrictions and to promote the bill of the same name, submitted for legislation by the Konfederacja political party. The premise of this bill was “to ban any forms of discrimination of the unvaccinated” [56]. The general hashtags “covid19”, “koronawirus”, and “szczepienie” (Eng. “vaccination”) were also popular, but the names reveal the true nature of this group: “konfederacja”, which is the political party strongly opposing vaccination mandate; “konstytucja”, referring to the opinion that the obligation to vaccinate would violate the Polish constitution; “niedzielskidodymisji” calling on the Minister of Health, Adam Niedzielski, to resign; and “gotowaniezaby” (Eng. “boiling frog”), a well-known apologue describing a frog being slowly boiled alive, which in this context means the fear of gradual limitation of personal freedom, e.g., by vaccination mandate. The remaining hashtags are “lextvn”, which refers to the government’s attempt to take control of TVN’s private television (some Twitter users interpreted this as a sham activity aimed at diverting attention from the planned introduction of the vaccination mandate), and “usa”, appearing in tweets describing the actions of American opponents of the vaccination mandate. The conducted hashtag review and tweet content review (described in Section 3.5) allowed us to classify the G2 group as opponents of the vaccination mandate.

Studying the behaviour of users of the G1 and G2 groups, we tracked the number of users in these groups throughout the entire study period. It is noteworthy that the G1 group (advocates) was more significant than the G2 group only at two time points: at the very beginning of the period under study, when the recommendation of the governmental medical council to introduce a vaccination mandate aroused great interest, and at the end, when the announced decision to introduce a vaccination mandate sparked a sudden increase in interest in this topic. Throughout the rest of the period, when the topic was not dominant in the media, the group of opponents (G2) was more frequent.

Studying the activity of users from the G1 and G2 groups, we analysed the number of tweets, retweets, and replies published on each day covered in the study by people belonging to each group. The types of user activity that were analysed can be defined as:

- Tweets, i.e., posting new content that includes the personal opinions of the author or links to news articles together with personal comments;
- Replies, i.e., direct responses to published tweets, interactions between Twitter users, an activity requiring engagement, exchange of opinions, dialogue, and comments;
- Retweets, i.e., forwarding a tweet or reply to reinforce its impact. This is a less engaging form of activity but crucial to understanding the reach of popular tweets.

**Figure 2:**
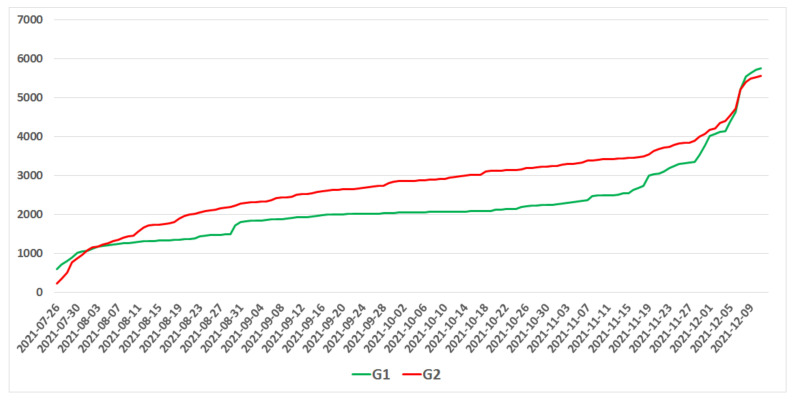
The size of the G1 (advocates) and G2 (opponents) groups throughout the analysed time period. The G1 group was larger than the G2 group only at two time points: at the very beginning of the period under study, when the recommendation of the governmental medical council to introduce a vaccination mandate aroused great interest, and at the end, when the decision to introduce a vaccination mandate was announced.

As shown in the Figure 2 above, Twitter users’ activity varied across the studied period. In the case of tweets and replies (activities requiring more involvement), G1 and G2 were balanced, while in the case of retweets (a more passive activity but reflecting the reach of influential posts), G2 was more often in the lead.

### 3.2. The Review of Social and Political Events Associated with G1 and G2 Members Activity

**Figure 3:**
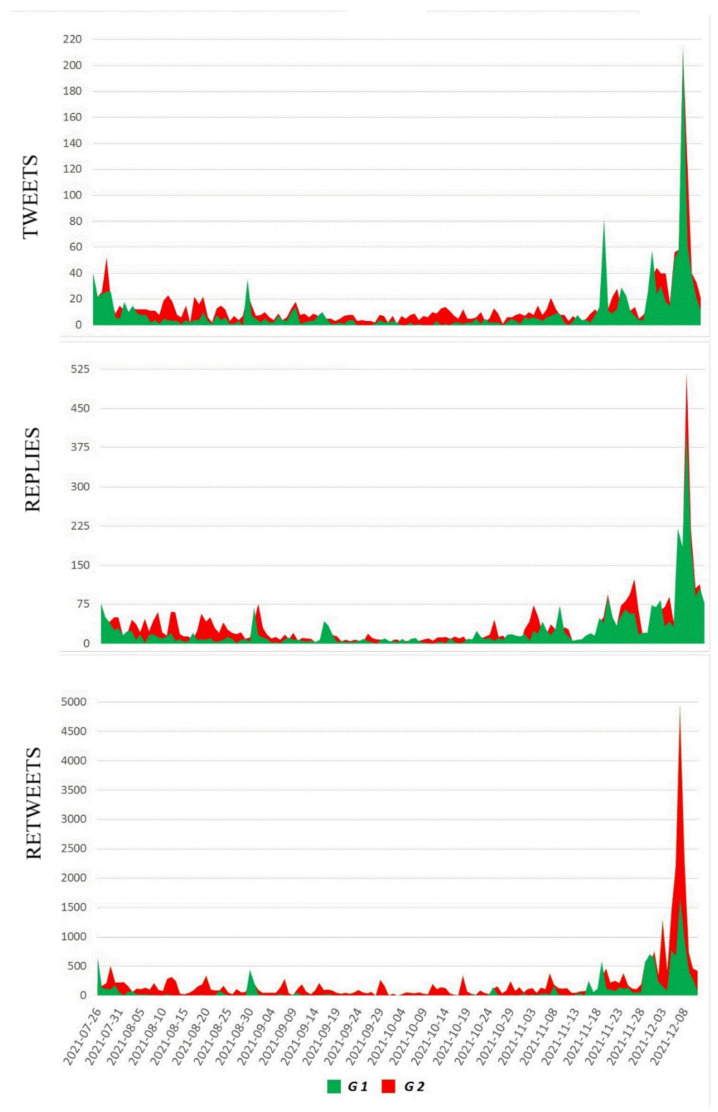
The number of tweets, replies, and retweets published daily by the members of the G1 and G2 groups. As for tweets and replies, both groups showed similar activity, whereas, when it comes to retweets, the G2 group (opponents) was more active.

Figure 3 shows that, during the studied time period, there were several dates on which a spike in activity in either the G1 group or the G2 group occurs. To answer the question of what social and political events most strongly influenced the activity of Twitter users, we analysed the content of tweets related to those days, and identified the key events associated with the ongoing debate. The results of this analysis are presented in Table 3.

### 3.3. The Measures of Correlation between Groups G1 and G2 Features and Epidemiological Data

**Figure 4:**
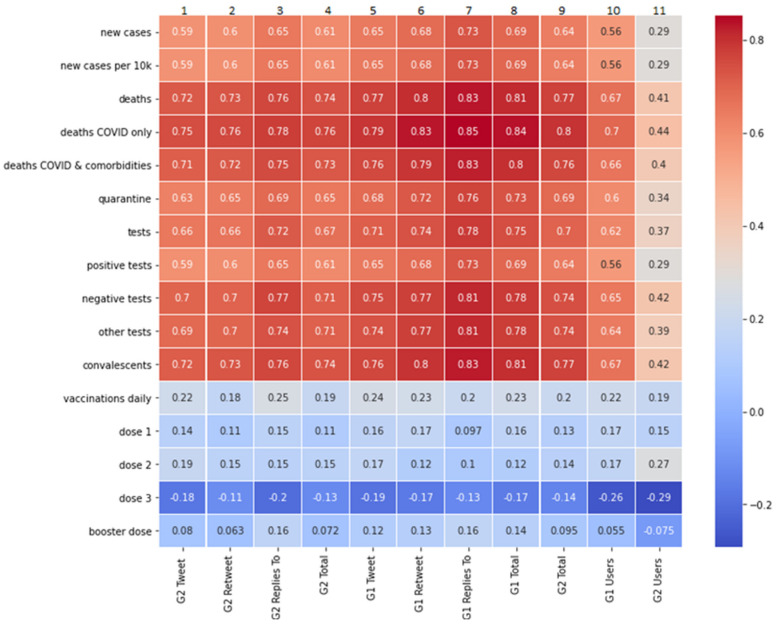
The measures of Pearson’s linear correlation between the COVID-19 epidemiological data and the number of tweets, retweets, and replies published in groups G1 and G2, as well as the size of the groups (labelled “GR1 Users” for the size of G1, and “GR2 Users” for the size of G2). The number of published tweets, retweets, and replies in both groups is strongly correlated with most epidemiological data, except for the vaccinations daily data.

Due to the fact that, in this analysis, we draw primarily upon the Pearson’ correlation coefficient method, when we discuss correlations below, we refer to this method by default. If, in some cases, we have used the Spearman’s rank correlation coefficient method, it will be clearly stated. Figure 4 presents the measures of linear correlation between the features of groups G1 and G2 and the daily number of vaccinations, new cases of COVID-19 infections, deaths caused by COVID-19, the number of people in quarantine, the number of tests performed, and the number of convalescents. Data for this analysis was obtained from the Polish Ministry of Health official statistics [40,41].

As shown in Figure 4, the number of published tweets, retweets, and replies in both groups is strongly (i.e., 0.6 and over) correlated with most epidemiological data. This relationship is most pronounced in relation to mortality statistics, negative tests performed, and the number of convalescents. Comparing the activity of groups G1 and G2, we note that the activity in G1 (in publishing retweets and replies) correlates more strongly with “negative tests” than the activity in G2. It can therefore be assumed that the negative test results confirm the belief of G1 members that vaccinations are effective, and that mandatory vaccination should be introduced.

The strongest correlations with epidemiological data in both G1 and G2 appeared in the number of replies, i.e., in Twitter activity, where high interaction between users of different opinions appears. The difference in favour of G1 versus G2 is revealed in the correlation of replies with new cases of disease (0.73 versus 0.65) and with “deaths from COVID & comorbidities” (0.83 versus 0.75).

The analysis of the correlation between the growing number of users in G1 and G2 groups and the epidemiological data leads to other conclusions. The increase in G1 abundance is strongly correlated with most epidemiological data (especially with “deaths COVID only” at 0.7), while G2 abundance is only weakly or moderately correlated with these data.

**Figure 5:**
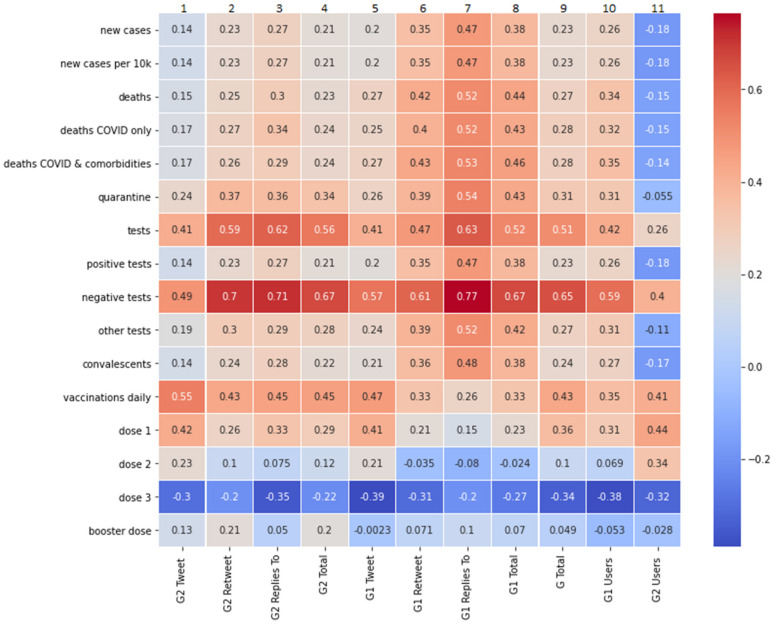
The measures of Spearman’s correlation between the COVID-19 epidemiological data and the number of tweets, retweets, and replies published in groups G1 and G2, as well as the size of the groups (labelled “GR1 Users” for the size of G1 and “GR2 Users” for the size of G2). The increase in the number of vaccinations performed is most strongly correlated with the increase in the number of tweets published in the G2 group (column 1).

When examining the correlation between the number of conducted vaccinations and the features of groups G1 and G2, we decided to use Spearman’s correlation coefficient (Figure 5). The Spearman’s coefficient, in this case, produces higher scores than the Pearson’s linear correlation coefficient, which means that the relationship is monotonic, not linear. The analysis shows that the increase in the number of vaccinations performed is most strongly correlated with the increase in the number of tweets published in the G2 group (column 1, value 0.55).

### 3.4. The Influential Users Regarding Betweenness Centrality

Table 4 shows the most influential users in the whole network, ranked by their betweenness centrality (BC) and numbered in the same order as in Figure 1. As we can see, nearly all of the important users were related either to group G1 or group G2. The betweenness centrality for each vertex is the number of shortest paths that pass through the vertex, i.e., the amount of influence a vertex has over the flow of information in a graph. The users that have high BC would have acted as key bridges within the social network. The column “Number of Followers” refers to the number of Twitter followers each user has. Only one of the influential nodes within the network belongs to an ordinary citizen who became an important bridge in the network. The other nodes belong mainly to the political figures and organisations: the account “a_niedzielski” belongs to the Polish Minister of Health, Adam Niedzielski; the accounts “konfederacja”, “pisorgpl”, and “_lewica” belong to the political parties involved in the pandemic debate; the account “piotr_schramm“ belongs to a well-known lawyer who is an opponent of mandatory vaccination; the account “morawieckim” belongs to the Polish Prime Minister, Mateusz Morawiecki; and “mz_gov_pl” is the official account of the Ministry of Health. Finally, “lukaszbok” is the account of Łukasz Bok, a popular internet journalist.

### 3.5. Analysis of the Most Popular Tweets

The next stage of our analysis was to identify the most popular tweets in the studied period, define their reach, and analyse their content in terms of their attitude to the vaccination mandate.

In order to identify the most popular tweets, we calculated the number of retweets received by tweets within the time frame of our study. Next, we made a ranked list of the 10 most popular tweets. To understand how people expressed their opinions, attitudes, and emotions toward the topic of mandatory vaccination, we manually conducted a sentiment analysis of the tweets, using the following coding categories: Category A (against mandatory vaccinations, or showing scepticism about the restrictions and threats related to the pandemic), Category F (in favour of mandatory vaccinations), and Category N (neutral or unidentified). Table 5 presents tweets included in the analysis, with the number of retweets assigned to them, the coding category, and the group they belong to (according to the clustering described above in Section 3.1). As can be seen, 8 out of 10 tweets on this list fall into the two largest groups, G1 and G2.

**Figure 6:**
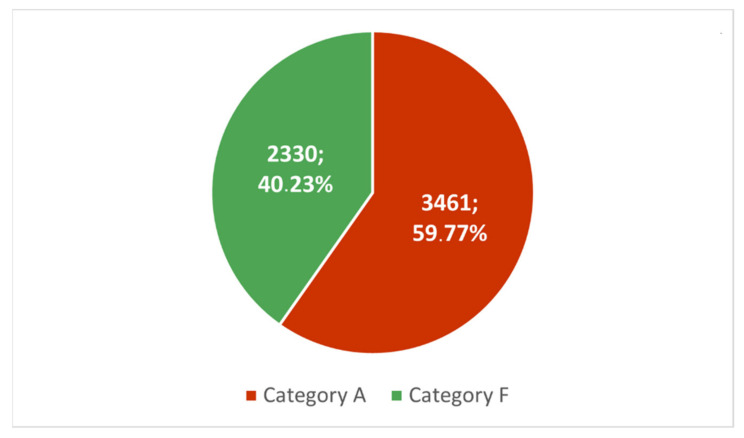
The summary of the number and percentage of retweets received by top-10 tweets is divided into Category A tweets (against vaccination mandate) and Category F tweets (in favour of vaccination mandate). Approx. 60% of the analysed content belongs to Category A (against mandatory vaccination).

As can be seen, there were no tweets labelled as “N” (neutral or unidentified) in this list. Instead, 7 out of 10 tweets were assigned to Category A (against mandatory vaccinations), and 3 out of 10 were assigned to Category F (in favour of mandatory vaccinations). Nevertheless, the two most popular tweets belonged to Category F. To reliably assess the true popularity of opposing positions among the Top-10 posts, we have summarised the number of retweets received by all tweets in a given category. The result is shown in Figure 6.

### 3.6. Correlations between Sentiment in the Top-10 Tweets and the Epidemiological Data

**Figure 7:**
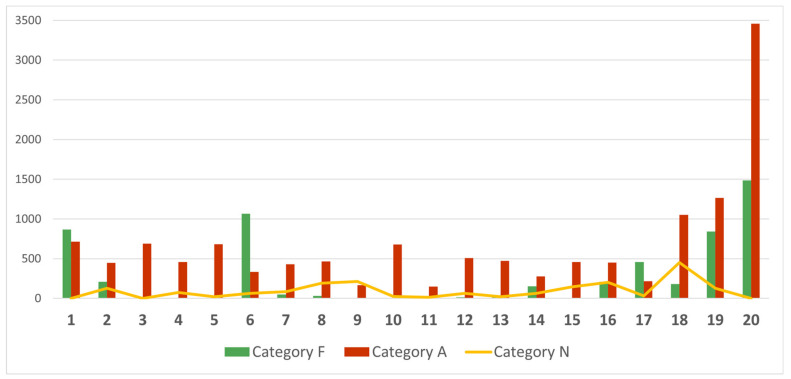
The summarised number of retweets received by top tweets identified for every week is divided into Category A (against vaccination mandate), Category F (in favour of vaccination mandate), and Category N (neutral or unidentified). For 17 out of 20 weeks, content belonging to Category A (against mandatory vaccination) was more popular.

To analyse the changes in Twitter users’ sentiments expressed in the top tweets, and to determine whether there were correlations between these changes and epidemiological data on COVID-19 in Poland, we divided our whole dataset into 20 partial datasets of one week each. For each of these datasets, we created the top-10 tweets ranked lists, using the same method as described in the previous section of this article. The summary of the number of retweets received by the top tweets in each studied week labelled as Category A (against vaccination mandate), F (in favour of vaccination mandate), and N (neutral or unidentified) is presented in Figure 7.

**Figure 8:**
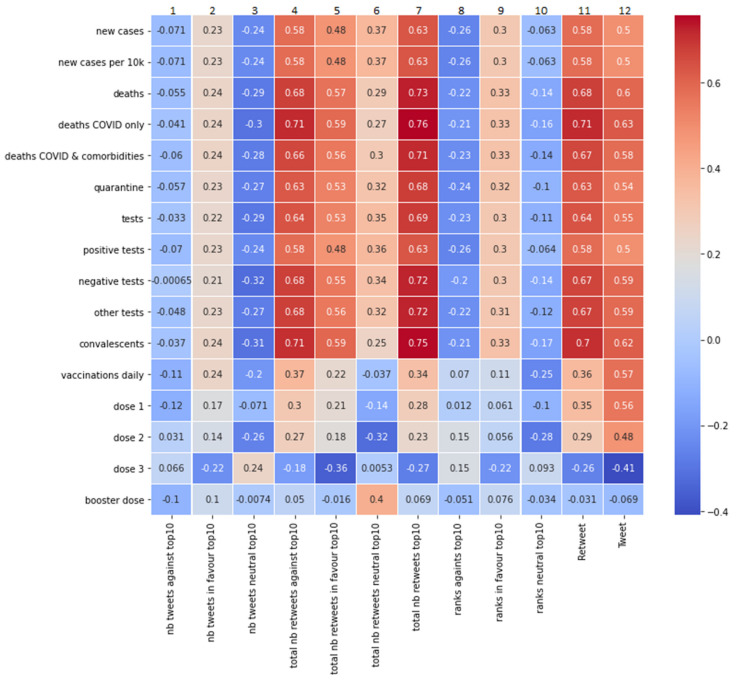
The measures of Pearson’s linear correlation between the COVID-19 epidemiological data and the sentiments expressed in the top-10 tweets. The total number of retweets for the top-10 tweets of all sentiment categories (column 7) correlates strongly with most medical data.

To analyse the correlations, we again used the Pearson’s correlation coefficient method. The results are presented in Figure 8. As we can see, the total number of retweets for the top-10 tweets of all sentiment categories (denoted as “total nb retweets top-10”, column 7, Figure 8) correlates strongly with most medical data. It is more strongly correlated than the total number of retweets in the entire sample, and thus is not limited to the top-10 tweets (column 11, Figure 8). This correlation occurs especially with the mortality rate (the greatest extent being “deaths COVID only”, at 0.71) and with the number of convalescents (0.70). This shows that both the increase in mortality rate and the increase in the number of convalescents are reflected in the debate on social media.

For comparative purposes, in addition to the analysis conducted using our basic method (correlations between epidemiological data and ranked lists taking into account the total sum of retweets for a given sentiment category, shown in Figure 8 as columns 4–6), we analysed correlations with tweet lists in which every tweet from the top-10 was treated the same, regardless of the number of retweets (columns 1–3 in Figure 8), and also correlations with tweet lists in which every tweet received a rank expressed in points depending on its position on the list (columns 8–10 in Figure 8). We also calculated the ratio for each of the considered methods by dividing the votes for, against, and neutral by the total number of votes. None of these additional methods showed any significant correlations, so we continued with the original method.

Comparing the correlations between epidemiological data and various categories of sentiment towards mandatory vaccination (presented in Figure 8: in column 4—tweets “against”, in column 5—tweets “in favour”, and in column 6—tweets “neutral”), we noticed that the sentiment “against” shows a slightly stronger correlation with epidemiological data than the “in favour” and “neutral” sentiments. This applies especially to the indicator “deaths COVID only” (correlated with the sentiment “against”, 0.71, and with the sentiment “in favour”, 0.59); the value of “negative tests” (correlated with the sentiment “against”, 0.68, and with the sentiment “in favour”, 0.55); and finally, the number of “convalescents” (correlated with the sentiment “against”, 0.71, and with the sentiment “in favour”, 0.59).

The situation is different for vaccination data (“vaccinations daily”). In this case, the correlation with retweets from the top-10 lists (columns 4–6, Figure 8) is only weak (0.37 or less). The strongest correlation of “vaccinations daily” occurs with the total number of tweets presenting new content (0.57; column 12, Figure 8).

### 3.7. Correlations between the Overall Network Metrics and the Epidemiological Data

**Figure 9:**
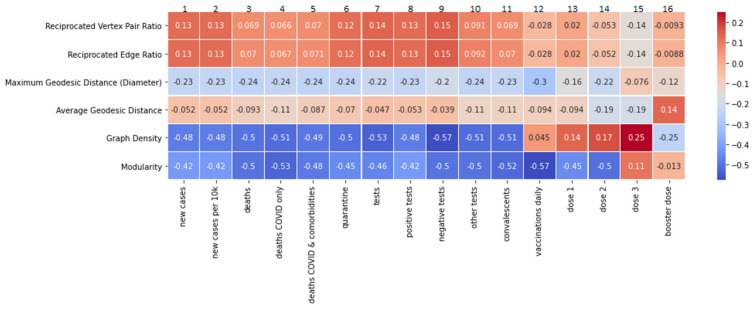
The measures of Pearson’s linear correlation between the COVID-19 epidemiological data and the overall network metrics. The graph density and modularity metrics are negatively correlated with most of the epidemiological data.

The last stage of our analysis was measuring the correlations between epidemiological data and overall network metrics. For this analysis, the same set of epidemiological data as in previous works was used. The network metrics, i.e., graph density, modularity, maximum geodesic distance, average geodesic distance, reciprocated vertex pair ratio, and reciprocated edge ratio, were calculated for each week of the study. The same samples as described in Section 3.6 were used. The results are presented in Figure 9.

In this analysis, we noticed the moderate negative correlations of graph density (GD) and modularity (MD) with the epidemiological data. This applies, in particular, to “new cases” (column 1: GD, −0.48; MD, −0.42); “deaths COVID only” (column 4: GD, −0.51; MD, −0.53); “negative tests” (column 9: GD, −0.57; MD, −0.5). Interestingly, in the case of “vaccinations daily”, the negative correlation appears only in the case of modularity (column 12, −0.57).

## 4. Discussion

RQ 1: What was the response on Twitter around the announcement of introducing mandatory vaccinations for COVID-19 in Poland, and what network shape emerged? What features characterised the key groups of advocates and opponents of mandatory vaccinations, and how was their activity correlated with official epidemiological data concerning the COVID-19 pandemic?

The overall features of the social network we identified in our analysis were examined by measuring the correlations between epidemiological data and overall network metrics tracked on a weekly basis. The observed negative correlations of graph density (see Figure 9) and modularity with the epidemiological data (with the exception of the “vaccinations daily” metric) show that the changes in the pandemic situation, either via the emergence of new epidemiological data or through other processes, increases the activity of the opposing groups on Twitter. This resulted in fragmentation of the debate and a decline in communication between polarised groups, as people became more distant from each other due to dissenting views and closing themselves in their own environments. Therefore, as can be seen in the analysed debate, the availability of epidemiological data, seemingly non-controversial, non-partisan, and non-emotional facts on the pandemic, does not lead to the convergence of opinions. Instead, it is connected with a deepening of interpretation differences. This observation is in line with earlier studies indicating that in a situation of highly polarised public debate, “science” or “facts” alone do not “solve” political controversies (nor do they change minds in a linear manner) [57]. A phenomenon known as “motivated numeracy” describes a situation where highly politically engaged persons in a partisan debate have higher competencies in interpreting numerical data, allowing them to rationalise the cognitive dissonance in the face of raw numerical data that contradicts their stance [58]. The lack of correlation with metrics such as “average geodesic distance” and “reciprocated vertex pair ratio” suggests that the disclosure of epidemiological data did not have any significant impact on collective intelligence emerging in the debate [52]. However, this does not mean that it did not influence the behaviour of the participants in the debate.

As described in Section 3.1, clustering with the use of the Clauset–Newman–Moore algorithm enabled the identification of 27 user groups that met the minimum criteria; among them were the two most important clusters, G1 and G2, in which almost 60% of the debates took place. The analyses of the most frequent hashtags used, and of social and political events affecting the debate, showed that G1 consists primarily of advocates of mandatory vaccination, and G2 consists of opponents of mandatory vaccination. Examining the influential users highlighted that the debate was strictly centred around political figures and organisations. The fact that the isolates group (G5), i.e., those who sent tweets that did not contain mentions, had a relatively low position in the performed study—unlike in other similar studies [42,45]—indicates that, in this case, users were very much involved in their subgroups, and that there is a large number of interactions (both within and between subgroups).

The results of the time series analysis of activities in both groups revealed large fluctuations in the number of tweets published over time, proving that the scale and intensity of discussions were strongly dependent on the current political statements, official health policy announcements, and media reports from other countries, examples of which we have cited in Section 3.2. The groups that were analysed were very similar in size, but the activity patterns of their members differed significantly. The group of advocates (G1) was larger than the group of opponents (G2) at only two time points of mobilisation: at the beginning of the analysed time period, when the idea of mandatory vaccination was proposed, and at the end of the analysed time period, when the vaccination mandate was officially announced to be introduced in March 2022. However, throughout most of the studied period, the activity of G2 was more consistent and greater in number overall. This leads to the conclusion that the advocates of mandatory vaccination were a group that was more difficult to mobilise for permanent engagement in the debate. This observation is in line with the view that contestation is one of the most important incentives motivating the participants of online debates [59]. Through collective contestation, participants develop autonomy and group identity, relatively speaking [60]. The observed relative balance between the two groups in tweets and replies was not the case with retweets, where a significant advantage of the opponents of the vaccination mandate was noticed, especially after 6 December 2021. The observed advantage of the G2 group in performing retweets indicates high emotional involvement resulting from the need to protest. The amount of original content (tweets) posted in both groups was comparable at the time, but the opponents, although less numerous, showed a greater commitment to promoting their position, and thus they were more visible on Twitter.

The correlation measurement, which we presented in Section 3.3, allowed us to observe some interesting phenomena. First, the increase in the number of vaccinations was strongly correlated with the increase in the number of tweets published in the group of vaccination opponents (G2). This may suggest that vaccination-critical tweets in the G2 group at a given time period were either counterproductive in discouraging vaccination, or that the members of the G2 group noticed a greater than the usual number of vaccinations (e.g., via social media announcements of people who vaccinated themselves), and this provoked group G2 to publicly reaffirm their stance. They often expressed the conviction that despite the increasing number of vaccinations, the number of cases of the disease was increasing, which strengthened their resistance to mandatory vaccination. The strongest correlations with medical data in both studied groups appeared in the publication of replies, i.e., when there is an interaction with users and an exchange of opinions with persons holding different views. Our analysis shows that in this type of interaction, the activity of advocates was more strongly correlated with published epidemiological data than the activity of opponents. Furthermore, the increase in the number of people in the group G1 (advocates) was strongly correlated with most epidemiological data, while the number of G2 (opponents) correlated with these data only to a weak or moderate degree. We conclude from this that advocates of mandatory vaccination, to a greater extent than opponents, paid attention to official epidemiological data, but were less motivated to remain active in the ongoing debate. Finally, measuring correlations with the Spearman method revealed that the activity of the advocate group in publishing retweets and replies was more strongly correlated with negative COVID-19 test results than the activity of opponents. Therefore, it can be hypothesised that the negative results of the tests convinced the people in the G1 cluster that vaccinations were effective, and that mandatory vaccination should be introduced.

RQ2: What was the general sentiment concerning mandatory vaccinations for COVID-19 expressed in the most popular tweets? How is this sentiment correlated with official epidemiological data concerning the COVID-19 pandemic?

The conducted analysis shows that the opinions of opponents were more visible and gained wider publicity than it would appear from the above-described activity of the G1 and G2 groups. In the top-10 ranking of tweets based on the entire sample, the vaccination opponents reached 59.77% of all corresponding retweets (Figure 6). Moreover, in the weekly analyses of top tweets, only 3 out of 20 of the surveyed weeks’ retweets made by advocates were more numerous (Figure 7). This is probably due to the mentioned attitude of protest and contestation expressed by opponents of the vaccination mandate. At the same time, however, we noticed that sentiments expressed in top-10 weekly rankings, although correlated with the epidemiological data (Figure 8), are correlated to a slightly lesser degree than the activity of members of the G1 and G2 groups (Figure 4). Therefore, it can be assumed that the opinions expressed in the most popular tweets are less dependent on actual epidemiological data, and more on the general world view of their representatives and on the opinions presented in the public debate.

We also showed that the relationship between “vaccinations daily” and the sentiments “in favour” or “against” the vaccination mandate is much weaker than in the case of other epidemiological data. It seems somewhat of a surprise that the decision to accept or not accept the vaccine is not related to supporting or not supporting the vaccination mandate (at least among Twitter users). When it comes to new COVID-19 cases, there is also a noticeable correlation, albeit at a more moderate level (0.58), with the anti-mandate sentiment. This shows that the increase in the incidence of the disease is associated with the greater intensity of the anti-mandate attitudes on Twitter. This might be due to the G2 members wanting to reaffirm their objection to the vaccination mandate in the face of epidemiological satiation that might legitimise this mandate. The above-described relationships occur to a greater extent in retweets than in tweets—which shows that epidemiological data has a slightly greater influence on general (more passive) interest in a topic than on publishing new content.

Strong views revealed in the majority of analysed tweets indicate the high intensity of the discussion and the high degree of involvement of the debate participants. What is interesting in the content analysis of the most popular tweets (see Table 5) is that, in some cases, strong anti-mandate stances are represented by persons declaring themselves as initially voting for the governing party (PiS). This indicates that the party’s electorate was either prone to such stances or heteronomous on the issue. In fact, the various ministers of the government were very ambivalent on the matter, some supporting the mandate (e.g., Minister of Health, Adam Niedzielski), others opposing it (e.g., Minister of Education, Przemyslaw Czarnek). This might be explained by the fact that PiS is described as a “textbook example” of a populist radical right (PRR) party [61], where PRR parties “are more likely to act on arguments with less scientific validity” [62]. However, since, in the health policy area, PiS is not a typical PRR party [63], its pandemic response policies might be disappointing to the anti-vaccination component of its constituency, where this specific part of the constituency became most vocal on social media.

Interestingly, the 2020 restrictions on legal forms of abortion (nominally by the ruling of the Polish Constitutional Tribunal, but, in popular opinion, considered as decided by the PiS leadership) were also invoked in ad hominem arguments—i.e., calling out the inconsistency of one’s stance—by the vaccine mandate supporters (“Forcing women to give birth to stillborn children is good, but the compulsion to vaccinate is not “because freedom of choice.” Hypocrite.”). This is an interesting interplay between the libertarian argument on personal liberty (of self-ownership), where the same rhetoric is being used to combat vaccination mandates on the one side and to criticise abortion restrictions on the other. Our research shows that the aforementioned tweet referencing this issue, emphasising inconsistency in the government’s stance on authority over women’s bodies, became the second-most retweeted within the studied period (another top-10 tweet stated: “Do you know why vaccinations “cannot” be compulsory? Because it is a personal matter, because of freedom? Well, because they also apply to men. It may be compulsory to give birth because it only affects women”). This was probably due to two factors at play. Firstly, the tweet was referring to another highly polarising and emotional issue that was still contentious among the Polish public opinion. Secondly, and simultaneously, the tweet resonated because it invoked particularly compelling framing [64] that was relevant for cultural cognition within certain wider narrative templates that invoke a set of recurring dramatic struggles and moral stakes [65].

The popularity of these tweets, alongside the prevalence of tweets criticising the government’s willingness to divert the vaccination mandates from its electorate, indicates the relevance of Twitter users’ discursive strategies that refer to the consistency and wider narrative frames of any given group. These invoke strong emotions, while at the same time relying on “reasoned persuasion”—where a logical component serves to trigger a new affective judgement—instead of just on “social persuasion”—where persuasion is merely based on announcing one’s stance on the matter to generate conformity [66]. Only one tweet out of the top 10 retweeted could strictly be categorised as “social persuasion”, with no elements of justification, context, or logical relations (“I declare that when @pisorgpl introduce compulsory or mandatory vaccinations for COVID, I will have nothing to lose and everything to gain, above all to maintain my dignity and freedom. Therefore, I will not forgive you for this! The anger will be great.”). None of the top 10 retweeted messages exemplified “bridging rhetoric”, i.e., an attempt at addressing the concerns and sensibilities of the group with an opposing view. All tweets with “reasoned persuasion” contained “bonding rhetoric”, i.e., were effectively addressed to the people with similar views to argumentatively reinforce them, and to rally around common sentiments [67]. This appears to be an aspect of the present and growing polarisation that is driven by partisanship at the level of opinion leaders’ discourse [68].

The contents of sampled anti-mandate tweets indicate lesser concern for the safety of vaccines (as in earlier studies [33]) and more interest in combating the perceived breach of personal liberties and discrimination through “sanitary segregation”. This might be due to the character of the discussion and the structure of the participating population. Surveys performed in the aforementioned studies used random selection to obtain the voices of the representative sample of the population, thus favouring giving personal reasons. The Twitter public debate, however, relies on the self-selection of (politically engaged) participants, and its context encourages the rationalisation or expression of preferences not based merely on personal feelings. The emotion of fear might be less prevalent in this context due to the expectation of reference to the public reason; i.e., robust reasoning (exclusionary to emotional motives) and general appeal (instead of personal/private interest) [69].

The content of sampled anti-mandate tweets is also clearly associated with conspiracy theories about pharmaceutical companies (“About 10 days ago, Austria made vaccination against C19 compulsory. Everyone wondered why “only” from February 2022. 10 days later, Omicron unexpectedly appeared, and Pfizer said it would take about 100 days to have a new vaccine”), and about a Jewish conspiracy to control Poland (“Then you have your Polin”, where “Polin” is the traditional name of Poland in Yiddish and Hebrew). This result is similar to the results obtained in studies performed in Spain [25,26].

In general, the evolution of the Twitter conversations within the analysed period shows that while at the beginning, tweets were more oriented toward information exchange, further exchanges were glowingly divergent instead of convergent. The progressing polarisation of the vaccination mandate debate was associated with the establishment of entrenched, strictly partisan narrative framing and with bonding rhetoric employed by analysed groups. This has led to the situation where the Twitter debate does not seem to result in any observable preference change among the debating parties, and in direct (functionally intended as persuasion) changes in behaviour that could be registered in epidemiological data. In some instances, it seems that the number of performed vaccinations was, counterintuitively, most strongly correlated, not with more pro-vaccination tweets, but rather with more anti-vaccination tweets. This shows that, at least in this particular case, the participation in Twitter public discourse (especially over time, as groups become more entrenched in their stances) can only be considered as a strict affirmation of positions, and not as an effective way of convincing the other side to preference change or even as a way of changing the behaviour of the non-debating public.

However, this result should not be interpreted in a way to suggest that pro-public health messaging on Twitter can be neglected. The lack of such messaging can have significant negative consequences by reducing the population’s health competency and, over time, by increasing vaccination hesitancy if the sole narrative visible is the one that negates vaccination benefits. It should be acknowledged, however, that Twitter as a platform specifically conductive for polarising affective debates and bonding rhetoric will have limited effectiveness in immediate public preference formulation on the basis of evidence-based knowledge. Still, this is not to say that such Twitter debate might not have an impact on public opinion over time by means of “knowledge creep” and through “decision by accretion” [70].

## 5. Conclusions

To the best of our knowledge, the present study is the first to address the analysis of a network focused on the debate concerning mandatory COVID-19 vaccination in the Polish-speaking community on Twitter. Our results are in line with previous research that revealed how Twitter played a crucial role in the spread of medical information and misinformation during the COVID-19 pandemic [37]. We also agree with the opinion presented in previous studies [25], that it would be appropriate to develop and implement public health surveillance programs focused on monitoring social networks in the field related to vaccination policy.

There are several new contributions that this study provides, mainly by deepening the research on the subject and relating it to the studies on political psychology and systemic approaches to public deliberation. It relates the analysis of Twitter activity to sets of relevant epidemiological data indicating multiple interesting correlations, sometimes expected and sometimes surprising. It was found that the shape of the network resembled a community model, as there was a range of users conversing with each other in different clusters. It was also found that a variety of accounts was influential and/or mentioned, ranging from politicians, political parties and organisations, media, public activists, and ordinary citizens. The groups of advocates and opponents of mandatory vaccination were active in a similar way, but the first group was more visible due to the large number of retweets. It was also established that the emergence of epidemiological data was not simply correlated with greater activity of those whose stances were seemingly supported by the data. For instance, a greater number of vaccinations was correlated with greater activity of vaccination mandate opponents. This opens up the field for future studies on the reasons for the popularity of this type of opinion, and the shape of public debate on vaccine-related topics. The key takeaways of our study can be summarised as follows:

- Those participants of the Twitter debates on vaccination who are most active do not represent the entirety of public opinion on the platform, although they can make a significant impact.
- The most impactful (most retweeted) posts—especially on the side of advocates of the vaccination mandate—are not those that merely announce bare emotions and “social persuasion”, but rather those that employ “reasoned persuasion” and wider narrative framing. This involves referring to more general principles and debates on other political issues.
- The vast majority of the debate participants are people who do not have a professional profile related to medicine or science. This applies to the opponents of vaccination mandate to a greater extent than to the advocates of vaccination mandate.
- Observing and analysing the most attention-capturing tweets may be helpful in crafting a better information policy concerning vaccines.
- Twitter debates on vaccines are dominated by “bonding rhetoric”—where communication is persuasive and addressed primarily to the like-minded. “Bridging rhetoric”—addressing emotion and valuing the sensitivities of the other side—is significantly underappreciated on Twitter. Government health policy could take into account “bridging rhetoric” to reach undecided people.
- Vaccination mandate advocates are consistently more numerous but less engaged and less mobilised to “preach” their own stances. Vaccination mandate opponents are vocal and more mobilised to participate, either as original authors or information diffusers.
- Vaccination mandate advocates are more numerous and, on occasion, are also able to mobilise. This, however, requires tweets that are particularly well crafted in terms of narrative framing.
- Systematic monitoring of the public debate on vaccines is essential not only in counteracting misinformation (debunking falsehoods, etc.,) but also in crafting evidence-based as well as emotionally motivating narratives.

The limitation of our study is the selected time period and the narrowing of the analysis to only one language. In addition, it has to be noted that the Twitter debate is not necessarily representative of the whole public view on the COVID-19 vaccination mandate.

## Figures and Tables

**Table 1:** Top 10 hashtags in the group G1.

Rank	Top Hashtags	Number of Occurrences
1.	covid19	1039
2.	szczepimysie (Eng. “we vaccinate”)	520
3.	koronawirus	379
4.	dworczyk	283
5.	pis	228
6.	szczepimysię (Eng. “we vaccinate”)	220
7.	covid_19	130
8.	polska (Eng. Poland)	118
9.	corona	105
10.	impfpflicht (Ger. “mandatory vaccination”)	105

**Table 2:** Top 10 hashtags in the group G2.

Rank	Top Hashtags	Number of Occurrences
1.	stopsegregacjisanitarnej (Eng. “stop sanitary segregation”)	2090
2.	covid_19	534
3.	koronawirus (Eng. “coronavirus”)	340
4.	lextvn	260
5.	konfederacja	220
6.	szczepienie (Eng. “vaccination”)	160
7.	konstytucja (Eng. “constitution”)	160
8.	usa	129
9.	niedzielskidodymisji (Eng. “Niedzielski to resign”)	126
10.	gotowanieżaby (Eng. “boiling frog”)	101

**Table 3:** Social and political events associated with tweets, retweets, and replies published in group G1 (opponents) and group G2 (supporters).

Date	Event	The Activity of Twitter Users in G1 and G2 Groups
26 July 2021	Medical Council of the Polish Prime Minister officially recommended the introduction of mandatory vaccinations.	Initially, an advantage of vaccination mandate supporters in tweets, replies, and retweets was observed. After two days, the opponents’ reaction brought them a considerable advantage in tweets, and a minor advantage in replies and retweets.
11 August 2021	“Lex TVN”: the government’s attempt to take control of TVN television was interpreted by some Twitter users as a sham activity aimed at diverting attention from the planned introduction of the vaccination mandate.	A considerable advantage of opponents in tweets, retweets, and replies was observed.
30 August 2021	A series of statements by the President of Poland and the Minister of Education declaring that the vaccination mandate will not be introduced.	There was a similar increase in supporters’ activity and opponents’ activity. It was observed mainly in publishing tweets and replies, less in publishing retweets.
09 September 2021	In the US, the president announced a vaccination mandate for federal officials and an obligation for companies to test employees. There was a lot of interest in these events in the Polish public sphere.	Similar supporters’ and opponents’ activity were observed, resulting in a similar number of tweets published, with a slight advantage over opponents. In the case of retweets, there was much more opponent activity.
15 September 2021	There was a large street demonstration of opponents of the vaccination mandate in Poland.	Reactions from vaccination advocates to the street demonstration were observed, calling for the introduction of mandatory vaccination. There was a significant advantage of supporters in the number of replies, a slight advantage of supporters in tweets, and a slight advantage of opponents in retweets.
14 October 2021	News from France: The Senate of the French Republic has spoken out against mandatory vaccination.	An advantage of opponents was visible mainly in the number of tweets published and, to a much lesser extent, in replies and retweets. No clear reaction from the supporters was observed.
19 November 2021	Austria introduces a vaccination mandate.	A high increase in the supporters’ activity mainly manifested in the number of tweets published.
29 November 2021	Statement by the President of Poland, Andrzej Duda: “I cannot imagine that Poland could introduce a vaccination mandate for COVID-19.”	A strong reaction from both supporters and opponents of mandatory vaccination. Supporters gained an advantage in the number of tweets published, while there was a balance in other areas.
06 December 2021	The government announced that the vaccination mandate would be introduced in March 2022 for several professional groups.	The greatest leap in G1 and G2 activity in the whole studied period. In the number of tweets and replies, supporters and opponents had similar values. In the number of retweets, the opponents gained a substantial advantage.

**Table 4:** Top 10 users ranked by betweenness centrality.

Rank	User Name	Betweenness Centrality	In-Degree	Number of Followers	Group
1.	a_niedzielski	47,020,459,613	2198	48,996	G2
2.	__lewica	35,299,154,045	1658	110,053	G1
3.	konfederacja_	32,537,643,789	1704	122,286	G2
4.	mz_gov_pl	23,730,195,090	1119	512,746	G1
5.	“citizen”	22,692,153,246	836	1336	G4
6.	pisorgpl	21,904,703,591	1415	271,723	G2
7.	piotr_schramm	21,141,628,593	1414	38,043	G2
8.	morawieckim	19,338,783,507	1361	426,536	G2
9.	lukaszbok	17,385,291,629	675	158,100	G1
10.	polsatnewspl	17,201,566,065	891	147,621	G1

**Table 5:** Sentiment analysis of top 10 retweeted posts in the analysed period. Classification categories: F, in favour of mandatory vaccinations; A, against mandatory vaccinations.

No.	Tweet	Group	Content Category	Date	Retweet Count
1.	*Do you think that vaccination against COVID-19 in Poland should be mandatory, as assumed by the @__Lewica project?*	G1	F	5 December 2021	1026
2.	*President: Forcing women to give birth to stillborn children is good, but compulsion to vaccinate is not “because freedom of choice.” Hypocrite.*	G4	F	30 August 2021	833
3.	*About 10 days ago, Austria made vaccination against C19 compulsory. Everyone wondered why “only” from February 2022. 10 days later Omicron unexpectedly appeared, and Pfizer said it would take about 100 days to have a new vaccine. When is it coming up?*	G2	A	29 November 2021	558
4.	*From March 1, 2022, COMPULSION of vaccinations for teachers, medics and uniformed workers under the threat of losing their jobs. Yes, in Poland.*	G2	A	7 December 2021	534
5.	*URGENT! From March 1, 2022, COMPULSION of “vaccinations” for teachers, medics and uniformed workers under the threat of losing their job. Then you have your Polin. Is it time to wake up or what else are bandits supposed to do to the Poles?*	G2	A	7 December 2021	531
6.	*I declare that when @pisorgpl introduce compulsory or mandatory vaccinations for COVID, I will have nothing to lose and everything to gain, above all to maintain my dignity and freedom. Therefore, I will not forgive you for this! The anger will be great.*	G3	A	6 December 2021	515
7.	*Do you know why vaccinations “cannot” be compulsory? Because it is a personal matter, because of freedom? Well, because they also apply to men. It may be compulsory to give birth because it only affects women.*	G1	F	6 December 2021	471
8.	*Complete lockdown in Austria and compulsory vaccination since February. In the Netherlands, the police shoot people. But remember, it’s all for your good.*	G2	A	20 November 2021	460
9.	*The obligation to vaccinate is the state’s liability for vaccine reactions. An obligation is constitutionally possible once it is proven that this restricts transmission. Thus, conscientious objection to unethical vaccinations is guaranteed by the constitution. @OrdoIuris will give legal aid.*	G2	A	7 December 2021	442
10.	*Italy, Trieste. Desperate and terrified workers who were refused to go to work due to lack of vaccination block the city’s port. Compulsory vaccine passports were introduced nationwide from October 15th. The same is happening with our neighbors in Lithuania.*	G2	A	18 October 2021	421

## Data Availability

The data presented in this study are available on request from the corresponding author.
